# Safety of median nerve electrical stimulation in disorders of consciousness: A systematic review and meta-analysis of randomized controlled trials

**DOI:** 10.1371/journal.pone.0324046

**Published:** 2025-07-31

**Authors:** Chunling Gu, Hongcai Shang, Yifan Kong, Mengqi Peng, Huiru Jiang, Shijia Wang, Xiaohong Wei

**Affiliations:** Key Laboratory of Chinese Internal Medicine of Ministry of Education, Dongzhimen Hospital, Beijing University of Chinese Medicine, Beijing, China; University of Cape Town Department of Paediatrics and Child Health, SOUTH AFRICA

## Abstract

**Background:**

Median nerve electrical stimulation (MNS) is a noninvasive treatment technique that can improve the brain functional activity of patients with disorders of consciousness (DoC) and promote their awakening. However, there is no research on whether MNS increases the incidence and severity of DoC complications. In this study, we evaluated the safety of MNS for the treatment of DoC.

**Methods:**

We searched relevant studies in PubMed, Cochrane, Web of Science (Medline), and Chinese databases, including CNKI, VIP, and WanFang Databases. For dichotomous outcomes, the pooled risk ratio (RR) with its 95% confidence interval (CI) was used as the effect estimate. We applied a fixed-effects or random-effects model to test the robustness of the forecast. A funnel plot was used to test for publication bias. Heterogeneity was evaluated using I². Subgroup analysis, sensitivity analysis were also performed. The quality of evidence was assessed using the GRADE approach. This study protocol has been registered in PROSPERO.

**Results:**

The results of the meta-analysis showed that MNS may not increase the incidence of complications and adverse events such as seizures, increased sympathetic activity, arrhythmia, nausea and vomiting, lethargy, pulmonary infection, intracranial hemorrhage or hematoma, and gastrointestinal bleeding in DoC patients. However, the methodological quality of most studies was poor, and accurate conclusions could not be drawn.

**Conclusion:**

MNS does not increase the incidence of DoC complications and adverse events, however, the quality of evidence for it’s safety is low and high-quality randomized controlled trials are needed to further confirm this conclusion.

## 1 Introduction

Disorders of consciousness (DoC) refer to a state in which the patient’s response to external stimuli is reduced or even where the patient is unresponsive, and which are generally caused by injury or dysfunction of the neural systems regulating arousal and awareness [1]. They are characterized by alterations in arousal and/or awareness, including the coma state, vegetative state/unresponsive wakefulness syndrome (VS/UWS) and the minimally conscious state (MCS) [2]. The causes leading to the DoC are various, involving diseases of multiple systems and organs, including brain diseases, endocrine and metabolic disorders, cardiovascular diseases, water and electrolyte imbalance, exogenous poisoning, physical and hypoxic damage [3]. For mild disorders of consciousness, the state of consciousness often recovers quickly after correcting the cause [4], while for severe disorders of consciousness, the treatment faces great difficulties. These patients are bedridden for long periods and have a low quality of life [5].

Currently, there are some methods to improve the level of consciousness in patients with DoC, such as pharmacological treatment, sensory stimulation, music therapy, electrical stimulation, hyperbaric oxygen therapy, acupuncture and moxibustion, and traditional Chinese medicine therapy, but their effects are limited [6,7]. Class II evidence was only found in two studies on amantadine and transcranial direct current stimulation [8,9], amantadine is only suitable for traumatic prolonged disorders of consciousness (pDoC), and it lacks efficacy for pDoC of other etiology [10].

Neuromodulatory stimulations are the most intriguing and hopeful alternative therapeutic approaches to DoC [11]. Median nerve stimulation (MNS) is a treatment that uses low-frequency electricity to electrically stimulate the skin in the anatomic area of a patient’s wrist or forearm where the median nerve is located [12]. Non-invasive peripheral neuromodulation therapy such as MNS has been used in clinical practice for awakening therapy for nearly two decades [13], since Yokohama et al. first reported the effects of MNS in patients with DoC [14]. MNS is one of the main techniques currently used for the treatment of DoC [15]. Liu et al. [12] reported that hyperbaric oxygen combined with MNS can improve the consciousness state of patients with consciousness disorders caused by brain injury and improve cerebral perfusion in patients with coma [16]. Xiong et al. [17] reported that MNS can improve the level of consciousness in patients with pDoC.

Currently, meta-analysis shows that MNS promotes recovery from DoC [18], and the studies on MNS mainly focus on comatose patients, with an emphasis on observing its role in pDoC [10]. However, in previous RCTs of MNS for the treatment of DoC, some studies have reported certain complications and adverse events of DoC, such as epileptic seizures, increased sympathetic activity, arrhythmia, nausea and vomiting, lethargy, pulmonary infection, intracranial hemorrhage or hematoma, and gastrointestinal bleeding [19–21]. Currently, there is no research on whether MNS increases the incidence and severity of these DoC complications and adverse events [22]. We therefore conducted a systematic review and meta-analysis of the published studies on the effects of MNS in patients with DoC to identify whether MNS may be a safe treatment for DoC.

## 2 Data and methods

We have described the methodology in more detail in a study protocol and have registered this study in PROSPERO (https://www.crd.york.ac.uk/PROSPERO/) (CRD42023470983). The Preferred Reporting Items for Systematic Reviews and Meta-Analyses (PRISMA) criteria were followed in the conduct of this systematic review and meta-analysis (S1 Table).

### 2.1 Selection criteria

Relevant studies were searched in six electronic databases from the date of their establishment to April 28, 2024, including PubMed, Cochrane, Web of Science (Medline), and Chinese databases, including CNKI, VIP, WanFang Databases, plus databases of ongoing trials (Clinical Trials.gov).

In this study, the interventions of interest include only MNS therapy; the outcomes of interest include only the incidence of adverse events and complications following the use of MNS therapy in patients with DoC. The search was limited to randomized controlled trials (RCTs) as study designs. We conducted a meticulous manual review of the reference lists in the retrieved articles to unearth any potentially eligible studies. There were no restrictions regarding language or publication status. The full search strategy is displayed in S2 Table.

### 2.2 Eligibility criteria

The articles had the following characteristics: (1) Type study: randomized controlled trials without any language or regional restrictions; (2) Participants: patients diagnosed with DoC by Coma Recovery Scale-Revised (CRS-R) or Glasgow Coma Scale (GCS), or coma due to cerebral hemorrhage or brain injury; (3) Intervention: MNS (a volar aspect of the forearm), regardless of the parameters used (type of current, frequencies, amplitudes, intensity) and the treatment duration, or comparison of using MNS with other control conditions such as sham stimulation, no stimulation, or any active control intervention; (4) Outcomes: the primary outcomes include the occurrence frequencies of seizures, increased sympathetic activity, arrhythmia, nausea and vomiting, lethargy, pulmonary infection, intracranial hemorrhage or hematoma, and gastrointestinal bleeding.

The exclusion criteria were as follows: (1) studies lacking a control group; (2) study types were case reports, animal experiments, meta-analyses, observational studies; (3) the incidence of adverse events or complications was not measured.

### 2.3 Measurement of the outcome variable

This systematic review and meta-analysis included eight outcome variables. The first outcome was the incidence of seizures. Epilepsy is a neurological disorder caused by abnormal electrical activity in brain cells, and seizures can lead to physical trauma, such as convulsions, loss of consciousness, and falls [23]. The second outcome is increased sympathetic activity. Neuroinflammation can trigger persistent sympathetic nerve excitation, causing the excitability and functional activity level of the sympathetic nervous system to be higher than the normal physiological state, thus leading to an increase in blood pressure and an enhancement of cardiac contractility [24]. Cardiac arrhythmias include irregular heartbeats, tachycardia or bradycardia. The severity of cardiac arrhythmias varies depending on their duration, heart rate, and impact on blood flow. These abnormalities can be either transient or chronic, and severe cardiac arrhythmias can lead to cardiac arrest [25]. Nausea refers to a state characterized by retching and/or persistent vomiting. Vomiting is a reflex action in which the contents of the stomach are expelled through the mouth; clinically, it is graded from 1 to 4 [26]. Pneumonia is an infection of the lung tissue. It is classified into community-acquired pneumonia, hospital-acquired pneumonia and pneumonia due to severe immunosuppression [27]. Intracranial hemorrhage refers to any bleeding within the intracranial vault, including the brain parenchyma and surrounding meningeal spaces. Following initial vessel rupture, the hematoma causes direct mechanical injury to the brain parenchyma. Symptoms may include headache, nausea, seizures, and focal or generalized neurologic symptoms [28]. Acute gastrointestinal bleeding presents as visible signs like hematemesis (vomiting blood), melena (black stools), or hematochezia (bright blood in stool); Chronic/occult bleeding is often detected through fecal occult blood tests or iron deficiency anemia; Bleeding of unknown origin describes recurrent cases where the source remains unidentified despite endoscopy and colonoscopy [29]. Lethargy consists of severe drowsiness in which the patient can be aroused by moderate stimuli and then drift back to sleep [30]. When a specific adverse event was defined differently in various studies, we referred to the most widely accepted and standardized definitions in the relevant field, which helped us aggregate the data.

### 2.4 Data extraction

Title and abstract screening and full-text screening were undertaken independently by two authors (CLG and YFK), and articles that did not meet the eligibility criteria were excluded, and the corresponding rationales for exclusion were documented. A standardized data extraction form was prepared in a Microsoft Excel 2016 spreadsheet and utilized. Two authors (SJW and HRJ) independently extracted relevant data using the following variables from the included studies: first author name, year of publication, country, study design, randomization method, sample size, type of DoC, intervention measures, and the occurrence frequencies of seizures, increased sympathetic activity, arrhythmia, nausea and vomiting, lethargy, pulmonary infection, intracranial hemorrhage or hematoma, and gastrointestinal bleeding. One investigator (MQP) conducted the data extraction from the included studies and another investigator (XHW) checked for consistency and any discrepancies. At the same time, discrepancies were resolved by a third author (HCS).

### 2.5 Assessment of risk of bias

Two independent investigators (CLG and YFK) assessed the risk of bias in light of the Cochrane Collaboration’s Risk of Bias tool [31]. The grades were rated as “low,” “high,” or “unclear” risk of bias based on the following items: random sequence generation, allocation concealment, incomplete data, blinding, selective reporting, and other sources of bias. Any differences were discussed and resolved with a third researcher (XHW).

### 2.6 Sensitivity analysis

To assess the robustness of the meta-analysis results, we conducted a sensitivity analysis for outcomes with high heterogeneity by sequentially removing each study and re-estimating the overall effect size.

### 2.7 Subgroup analysis

If the sensitivity analysis fails to adequately explain the observed heterogeneity, we conducted subgroup analyses to explore the potential sources of variability.

### 2.8 Assessment of quality of evidence

Two independent investigators MQP and SJW used the GRADEpro GDT system to assess the evidence quality of the outcomes [32]. This system comprises five aspects: study limitations, inconsistency, indirectness, imprecision, and publication bias. The quality of the evidence was categorized as high, moderate, low, and very low. Any differences were discussed and resolved with a third researcher (XHW).

### 2.9 Statistical analysis

Data were aggregated and analyzed using Review Manager (version 5.3). For dichotomous outcomes, the pooled risk ratio (RR) with 95% CI served as the effect estimate. Heterogeneity was assessed using the I-squared statistic, with thresholds of 25%, 50%, and 75% indicating low, moderate, and high heterogeneity, respectively [33]. If the I² < 50% and p > 0.1, heterogeneity was deemed low, and a fixed-effects model was applied for meta-analysis. If the I² > 50% or p ≦ 0.1, significant heterogeneity was identified; in these cases, sensitivity and subgroup analyses were performed with Stata 15 to investigate potential sources of heterogeneity. When subgroup analyses failed to resolve heterogeneity, a random-effects model was used. Combined risk estimates were displayed in forest plots, and publication bias was evaluated via funnel plots when ≥ 10 studies were included in a group [34].

### 2.8 Ethical approval and consent

Ethical approval and consent to participate did not apply to this study because this study is based exclusively on published literature.

## 3 Results

### 3.1 Study selection

**Fig 1:**
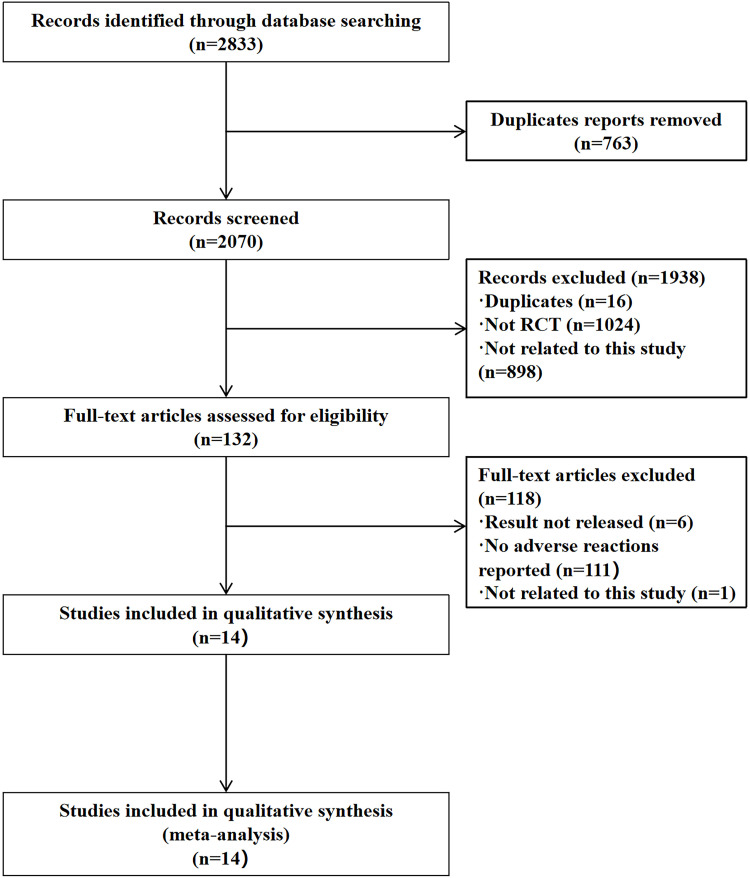
Study flow diagram. No adverse reactions reported: the outcomes of interest were not measured.

The PRISMA statement flow diagram is shown in Fig 1. A total of 2833 records were part of the initial database search (PubMed: 177, Web of Science: 306, CENTRAL: 1830, CNKI: 147, VIP: 178, Wanfang: 195), and we excluded 763 duplicate records. After filtering the titles and abstracts to exclude irrelevant articles, we found 132 articles that met the topic of interest. Of them, 118 articles were excluded, and 14 studies from the database searches were included in the systematic review and meta-analysis. A list of excluded full-text reports is provided in S3 Table.

### 3.2 Study characteristics

A total of 1729 patients were included in the 14 studies, 869 in experimental groups and 860 in control groups. Twelve studies were in patients with coma, and 2 studies were in patients with DoC. The experimental groups in 12 studies were “Routine therapy+MNS/RMNS (Right Median Nerve Electrical Stimulation)”, and the control groups were “Routine Treatment”. Two studies were “Routine treatment+Acupuncture+RMNS”, and the control groups were “Routine treatment+Acupuncture”. These studies were published between 2015 and 2023, and all the patients involved were from China. S4 Table shows the detailed characteristics of the 14 studies that underwent final analysis herein.

### 3.3 Risk of bias in the analyzed studies

**Fig 2:**
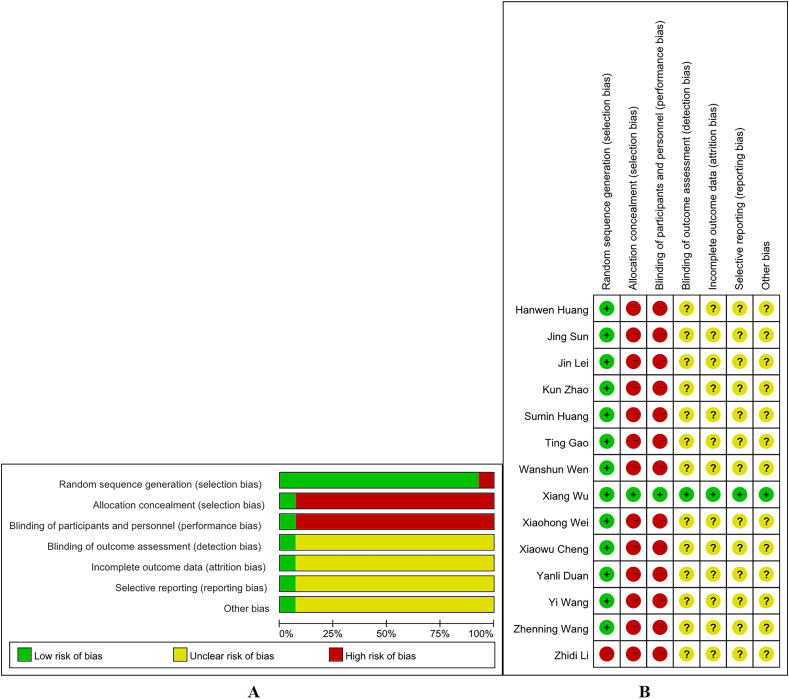
Risk of bias in the analyzed studies. (A) Risk of bias graph. (B) Risk of bias summary.

We investigated the risk of bias for all articles included in our analysis (Fig 2 A-B). Most studies were randomly divided into an experimental and control groups. The randomization methods of most studies were classified as having a low risk of bias because they used random number tables for grouping. The risks of “blinding of participants and personnel” and “allocation concealment” are high in most studies. In most studies, the aspects of “selective reporting”, “blinding of outcome assessment”, and “other biases” were evaluated as having an unclear risk of bias.

### 3.4 Risk of MNS with Doc

#### 3.4.1 Seizure.

**Fig 3:**
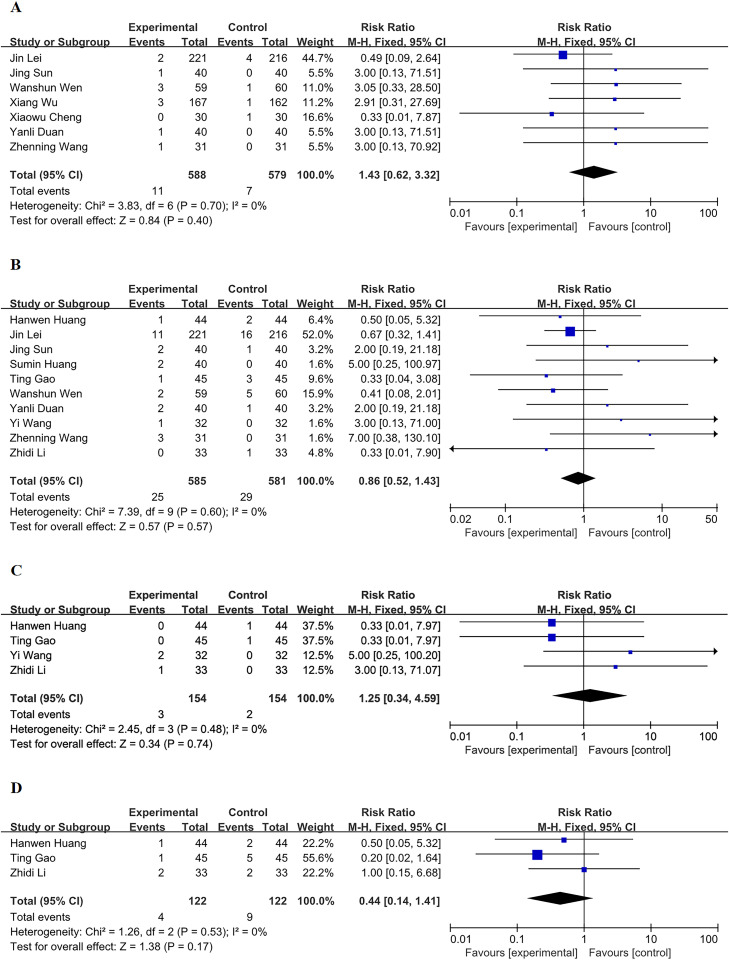
Forest plots for seizures, increased sympathetic nerve activity, arrhythmia, nausea and vomiting. (A) Forest plot for seizure. (B) Forest plot for increased sympathetic activity. (C) Forest plot for arrhythmia. (D) Forest plot for nausea and vomiting.

The MNS did not have a statistically significant impact on seizure events based on 7 studies reporting on this outcome (RR:1.43, 95%CI 0.62–3.32, p = 0.40), and there was no evidence of significant heterogeneity (I^2^ = 0%, p = 0.70, Fig 3A).

#### 3.4.2 Increased sympathetic activity.

The MNS did not have a statistically significant impact on increased sympathetic activity events based on 10 studies reporting on this outcome (RR:0.86, 95% CI 0.52–1.43, p = 0.57), and there was no evidence of significant heterogeneity (I^2^ = 0%, p = 0.60, Fig 3B).

#### 3.4.3 Arrhythmia.

Four studies reported arrhythmia. The MNS did not have a statistically significant impact on arrhythmia (RR:1.25, 95% CI 0.34–4.59, p = 0.74), and there was no evidence of significant heterogeneity (I2 = 0%, p = 0.48, Fig 3C).

#### 3.4.4 Nausea and vomiting.

Three studies reported nausea and vomiting. The MNS did not have a statistically significant impact on nausea and vomiting (RR 0.44, 95% CI 0.14–1.41, p = 0.17), and there was no evidence of significant heterogeneity (I^2^ = 0%, p = 0.53, Fig 3D).

#### 3.4.5 Lethargy.

**Fig 4:**
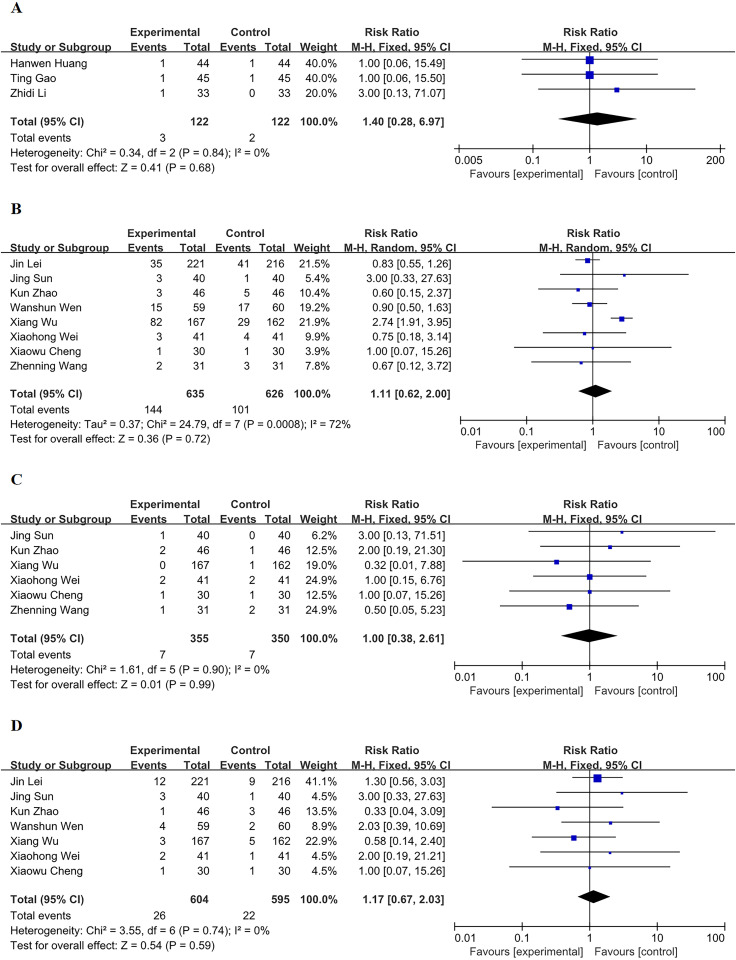
Forest plots for lethargy, pulmonary infection, intracranial hemorrhage or hematoma, and gastrointestinal bleeding. (A) Forest plot for lethargy. (B) Forest plot for pulmonary infection. (C) Forest plot for intracranial hemorrhage or hematoma. (D) Forest plot for gastrointestinal bleeding.

The MNS therapy did not have a statistically significant impact on lethargy based on 3 studies reporting on this outcome (RR: 1.40, 95% CI 0.28–6.97, p = 0.68), and there was no evidence of significant heterogeneity (I^2^ = 0%, p = 0.84, Fig 4A).

#### 3.4.6 Pulmonary infection.

Eight studies reported pulmonary infection. The MNS did not have a statistically significant impact on pulmonary infection (RR 1.11, 95% CI 0.62–2.00, p = 0.72), but substantial statistical heterogeneity was observed (tau^2^ = 0.37, I^2^ = 72%, p < 0.01, Fig 4B).

#### 3.4.7 Intracranial hemorrhage or hematoma.

The MNS did not have a statistically significant impact on intracranial hemorrhage or hematoma based on 6 studies reporting on this outcome (RR:1.00, 95% CI 0.38–2.61, p = 0.99), and there was no evidence of significant heterogeneity (I^2^ = 0%, p = 0.90, Fig 4C).

#### 3.4.8 Gastrointestinal bleeding.

The MNS did not have a statistically significant impact on gastrointestinal bleeding based on 7 studies reporting on this outcome (RR:1.17, 95% CI 0.67–2.03, p = 0.59), and there was no evidence of significant heterogeneity (I^2^ = 0%, p = 0.74, Fig 4D).

### 3.5 Assessment of publication bias

**Fig 5:**
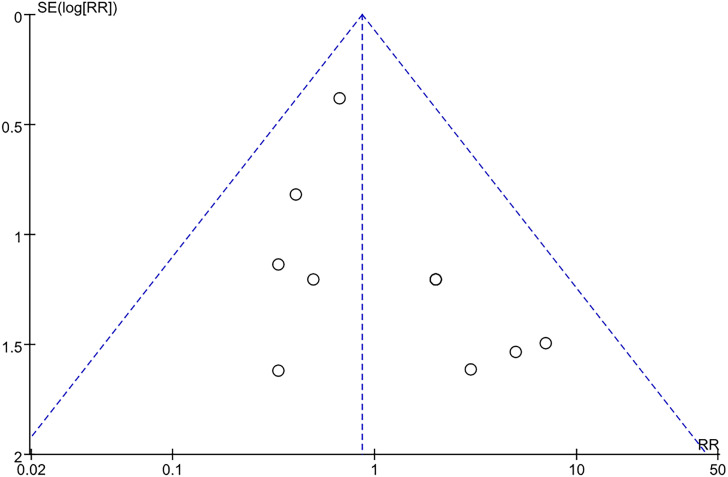
Funnel plot for increased sympathetic activity.

Ten studies were included in meta-analysis of increased sympathetic activity, so a funnel plot was used to detect publication bias (Fig 5).

For increased sympathetic activity, publication bias results: the funnel plot showed symmetry, indicating no evidence of publication bias.

### 3.6 Sensitivity analysis

**Fig 6:**
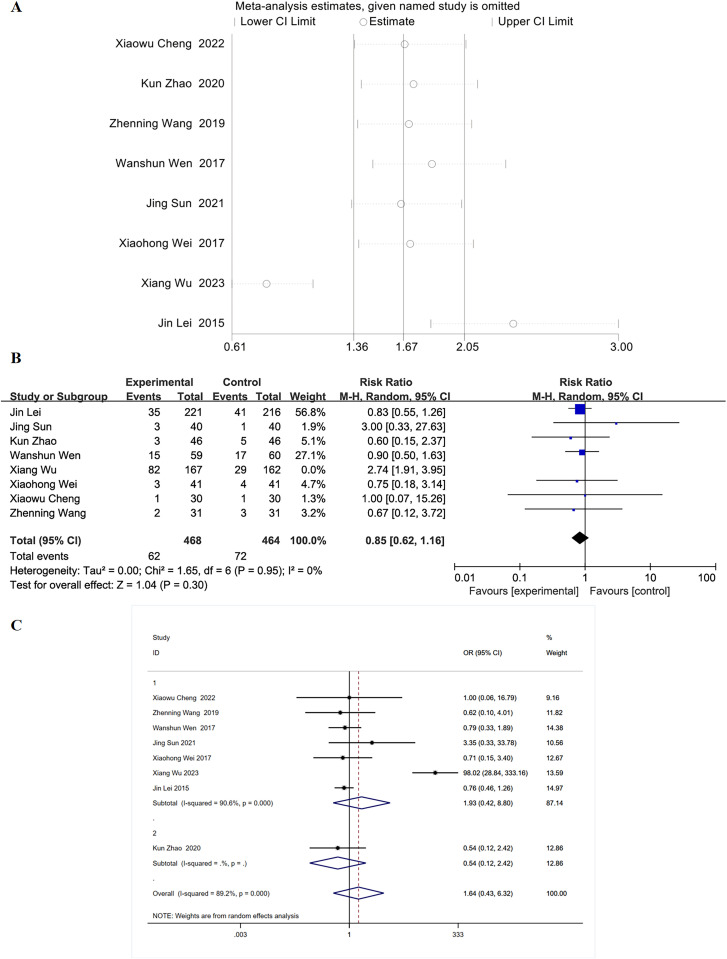
Sensitivity analysis, forest plot and subgroup analysis of pulmonary infection. (A) Sensitivity analysis of pulmonary infection. (B) Forest plot for pulmonary infection. (C) Sub-group analysis of the outcomes of pulmonary infection according to the intervention measures; Group 1: Routine treatment + RMNS vs Routine treatment; Group 2: Routine treatment + Acupuncture + RMNS vs Routine treatment + Acupuncture.

Because of the high heterogeneity of the outcome indicators for pulmonary infection, we conducted a sensitivity analysis to identify the potential sources of heterogeneity and examine the stability of the results. The results showed that the study by “Xiang Wu 2023” was the source of the high heterogeneity (Fig 6A). After excluding this study, a meta-analysis was performed using the random effects model, and the results showed that I^2^ = 0%, P = 0.30 (Fig 6B). We believe that the reason for the high heterogeneity of the results was that the study by Xiang Wu et al. had a larger sample size compared to other studies, the differences in sample size are potential sources of high heterogeneity.

### 3.7 Subgroup analysis

Among the indicators of pulmonary infection, the intervention measures in the study by “Kun Zhao 2020” are different from those in other studies. The intervention measures in the study by “Kun Zhao 2020” involve “Routine treatment + Acupuncture + RMNS” and “Routine treatment + Acupuncture”, while other studies compare the therapeutic effects between “Routine treatment + RMNS” and “Routine treatment”. Therefore, we conducted a subgroup analysis according to the intervention measures. The results of the subgroup analysis indicate that there is still a high level of heterogeneity (I^2^ = 89.2%, P = 0.00), it is suggested that the study by “Xiang Wu 2023” may be a potential source of high heterogeneity (Fig 6C).

### 3.8 Quality of evidence

The GRADE system was used to evaluate the evidence quality of outcomes (S5 Table). The quality of evidence was low for most studies due to unclear blinding, inadequate allocation concealment, and wide 95% CIs. In addition to these downgrades, the quality of evidence for pulmonary infection is very low due to high heterogeneity.

## 4 Discussion

As an effective restorative therapy for DoC, nerve stimulation has received increasing attention [35]. MNS is a peripheral stimulation that acts on the median nerve. Arousal is considered mostly related to the ascending reticular activating system (ARAS). Stimulation of the median nerve can stimulate the electrophysiological activities of neurons at all levels through the ascending fibers of the spinal nerves, cervical cord, brain stem, thalamic-cortex region, and trigger a series of central excitatory effects, activate the inhibited neurons and ARAS, and thus promote the recovery of consciousness [36]. Studies have shown that MNS can induce an up-regulation of orexin-A and OX1R expression in the prefrontal cortex of coma rats induced by traumatic brain injury [37]. For more than 20 years, MNS has been used as an inexpensive and non-invasive treatment method to promote the awakening of patients with DoC, which can reduce the disability rate and improve the prognosis of patients [38,39]. However, in previous RCTs of MNS for the treatment of DoC, complications and adverse events of DoC such as seizures, increased sympathetic activity, arrhythmia, nausea and vomiting, lethargy, pulmonary infection, intracranial hemorrhage or hematoma, and gastrointestinal bleeding have all been reported. Does MNS increase the incidence and severity of these DoC complications and adverse events? This systematic review aims to evaluate the safety of MNS in patients with DoC and assess whether MNS increases the incidence and severity of DoC complications and adverse events.

After conducting a systematic review and meta-analysis of the 14 included studies [40–53], among the included studies, DoC were mainly caused by severe brain injuries, including intracerebral hemorrhage and craniocerebral injury. We found that MNS may not increase the incidence of complications and adverse events such as seizures, increased sympathetic activity, arrhythmia, nausea and vomiting, lethargy, pulmonary infection, intracranial hemorrhage or hematoma, and gastrointestinal bleeding in patients with DoC. However, the methodological quality of most studies was poor, and the quality of evidence was low. Therefore, we could not draw definite conclusions.

The RCTs of DoC included in this study have many problems in aspects such as study design, implementation process, and result reporting. During the implementation process, randomization and blinding methods are not rigorous. Most trials only mention the random allocation of patients using a random number table, but the random sequence and allocation details are not disclosed. Most studies did not mention whether blinding was implemented for patients, doctors, and outcome assessors. Since it involves neuroelectrical stimulation, it is difficult to apply blinding to patients and doctors. Therefore, we judged that most studies had a high risk of bias in terms of “blinding of participants and personnel” and “allocation concealment”. As it is not mentioned whether blinding was applied to statisticians, we judged that the bias risk of “blinding of outcome assessment” was unclear. In terms of “selective reporting”, by carefully checking the method and result sections of each study and comparing them with the pre-registered research protocols, we judged whether there was a problem with selective reporting. However, only a few studies have pre-registered research protocols, and most studies did not pre-register their research protocols, therefore, we believe that most studies have an unclear risk of bias in selective reporting. In terms of “incomplete outcome data”, since it is not mentioned whether any data were omitted from the post-treatment evaluation, nor the reference procedures followed, we believe that most studies have an unclear risk of bias. “Other bias”, which is used to note bias occurring due to any additional problems that were not covered by the first six domains, such as deviations from the study protocol, giving intervention before randomization, inappropriate administration of an intervention, contamination due to drug pooling among participants, insufficient delivery of intervention, and fraud [54]. Most of the included studies did not provide sufficient details, so we classified “other bias” as an unclear risk of bias. In terms of result reporting, there are differences in the reporting content and methods of adverse events and complications among different studies, regarding the reporting content, the degree of detail in symptom descriptions varies among studies, and some studies do not grade the severity of symptoms, so we cannot study whether MNS will aggravate the severity of DoC complications and adverse events, for the occurrence time of adverse events and complications, the records of some studies are not accurate and detailed enough, and some studies even do not report the occurrence time of adverse events and complications. Outcomes of pulmonary infections are highly heterogeneous, indicating the lack of robustness of the results. The results of the sensitivity analysis showed that differences in sample size were potential factors contributing to high heterogeneity, either an excessively large or small sample size may lead to increased variability in the results and a risk of bias.

Future MNS RCTs for the treatment of DoC should have a high-quality methodology, particularly regarding blinding, randomization, and incomplete outcome data. For example, the random sequence should be revealed and scientific randomization techniques, like computer-generated random numbers, random number tables, or randomization software, should be used in the randomization design. In the blinding design, it can be considered to cover the patient’s forearm during the stimulation process or conduct sham stimulation. Blinding should be implemented for outcome assessors and data analysts to reduce the interference of subjective factors on the research results. In the inclusion and exclusion criteria, standardized assessment methods with high-quality evidence should be used. A systematic review conducted by the American Congress of Rehabilitation Medicine recommends the use of the Coma Recovery Scale-Revised (CRS-R), Wessex Head Injury Matrix, Sensory Modality Assessment and Rehabilitation Technique, Western NeuroSensory Stimulation Protocol, Disorders of Consciousness Scale and Sensory Stimulation Assessment Measure for clinical practice [55–57]. In terms of sample size, the sample size should be calculated reasonably to ensure that the study has sufficient statistical power. In terms of research reporting, patient characteristics (age, disease duration, disease stage, habits), data on the continuous steps from coma to full consciousness, and detailed information on intervention and control measures should be recorded and disclosed. This includes clarifying the placement location of electrode patches, operation procedures, the basis for selecting stimulation parameters and adjustment methods, as well as the frequency and course of the intervention. Since all patients are in a comatose state, the incidence and severity of DoC complications and adverse events should be rigorously examined. These complications include but are not limited to the risk of seizures, increased intracranial pressure, skin damage, skin abrasions, pressure ulcers, increased sympathetic nerve activity, tinnitus, significant changes in respiratory rate, heart rate, blood pressure, and/or blood oxygen saturation, heart diseases, pneumonia, hypertonia, musculoskeletal disorders, and pain [21]. Future studies should observe whether MNS increases the incidence and severity of DoC complications and adverse events. In future RCTs of MNS for the treatment of DoC, it is necessary to refer to relevant international guidelines or consensus. Researchers should clearly define the scope of adverse events, unify reporting standards, use standardized forms or report templates to record adverse events, and list in detail information such as the name of the adverse event, occurrence time, severity, treatment plan, and disease outcome. Moreover, it is essential to strengthen the training of researchers in adverse event reporting. The training content should cover knowledge about the definition, classification, judgment criteria, reporting procedures, and requirements of adverse events. Long-term follow-up studies are necessary, and future research must examine the long-term effects of MNS in greater detail rather than only the short-term ones [58]. In addition, there is a lack of research on special patient subgroups. It is necessary to pay attention to subgroup studies of special populations (such as children, the elderly, and patients with rare etiologies) among patients with DoC. This is of great significance for a deeper understanding of the pathogenesis of DoC and for optimizing the MNS treatment plan and its effectiveness.

In the included studies, Yanli D et al. [40], Kun Z et al. [43], Hanwen H et al. [44]_,_ and Ting G et al. [51] used acupuncture at Neiguan (PC 6) to treat DoC, Hanwen H and Ting G used electrical stimulation at Neiguan (PC 6) to treat DoC. They used asymmetric bidirectional pulse continuous wave to treat the slight contraction of patients’ fingers on both sides as the adjustment basis, and GCS scores of patients were improved after acupuncture treatment.

Acupuncture, one of the oldest non-drug therapies, is the practice of inserting fine needles into acupuncture points to treat diseases [59,60]. Some scholars have conducted data mining on ancient Chinese medical literature and proposed that researchers should pay attention to the use of Neiguan (PC 6) in the treatment of DoC [61]. Neiguan (PC 6) is one of the commonly used points in the hand Jueyin pericardium channel and is located in the middle of the transverse stripe of the wrist. Studies have shown that the median nerve is located at the Neiguan (PC 6) [62]. Stimulation of Neiguan (PC 6) activates the median nerve [63], brain stem reticular structure, and hypothalamus, relieve the inhibition of the non-specific ascending excitation system, and reduce the degree of coma [64]. In summary, it is suggested that electrical stimulation of Neiguan (PC 6) may be an additional method to awaken patients by stimulating the median nerve.

### 4.1 Limitations

Our research has some limitations. First, most of the clinical trials included in this study were conducted in China and published in Chinese, which limits our ability to summarize conclusions. Secondly, most of the included studies were of poor methodological quality, high overall bias risk, and low evidence quality, makeing it impossible to draw definitive conclusions. In addition, we found several randomized controlled trials in the clinical trial protocol database, but there is no possibility of obtaining the publication date of the study or providing us with the original data.

## 5. Conclusion

MNS does not increase the incidence of DoC complications and adverse events, however, the quality of evidence for its safety is low. Future research should include rigorous methodological trials that monitor long-term follow-up outcomes and explicitly report adverse events and complications using standardized criteria. Randomized clinical trials can best inform clinicians and patients about treatment safety only when they provide comprehensive information about adverse events and complications reporting.

## Supporting information

S1 Table PRISMA 2020 checklist. (DOCX)

S2 Table Search strategy used for the electronic databases. (DOCX)

S3 Table List of excluded studies. (XLSX)

S4 Table Characteristics of the included studies. (DOCX)

S5 Table Quality of evidence. (DOCX)

## Abbreviations

CI: confidence interval
DoC: disorders of consciousness
MCS: minimally conscious state
MNS: median nerve stimulation
pDoC: prolonged disorders of consciousness
RCT: randomized controlled trial
RR: risk ratio
UWS: unresponsive wakefulness syndrome;
VS: vegetative state
