# Supplementary material for: Safety of median nerve electrical stimulation in disorders of consciousness: A systematic review and meta-analysis of randomized controlled trials
Source: PLoS One. 2025 Jul 31;20(7):e0324046. doi: 10.1371/journal.pone.0324046 (PMC12312889; doi:10.1371/journal.pone.0324046)
Supplement: S2 Table — (DOCX) [file pone.0324046.s002.docx]

**S2 Table. Search strategy**

The search strategy was constructed for PubMed and amended accordingly for other electronic databases. No restrictions on language or publication date were applied. The reference lists of the retrieved reports were manually searched for other non-identified relevant reports. Clinicaltrials.gov was searched for ongoing or completed unpublished trials. Date run: 28/04/2024. The search syntax and search string for each electronic database is presented below:

| **Data bases** | **Search strategy** |
| --- | --- |
| PubMed | (("Median Nerve"[Mesh])OR(((Median Nerves[Title/Abstract])OR(Nerve,Median[Title/Abstract]))OR(Nerves,Median[Title/Abstract])))AND(("Persistent Vegetative State"[Mesh])OR(((((((((((((((((((((((((((((((((((((((((((((((((((((((((((((((((((((((((PVS(Persistent Vegetative State[Title/Abstract]))OR(PVSs(Persistent Vegetative State[Title/Abstract])))OR(Vegetative State,Persistent[Title/Abstract]))OR(Persistent Vegetative States[Title/Abstract]))OR(State,Persistent Vegetative[Title/Abstract]))OR(States,Persistent Vegetative[Title/Abstract]))OR(Vegetative States,Persistent[Title/Abstract]))OR(Persistent Unawareness State[Title/Abstract]))OR(Persistent Unawareness States[Title/Abstract]))OR(State,Persistent Unawareness[Title/Abstract]))OR(States,Persistent Unawareness[Title/Abstract]))OR(Unawareness State,Persistent[Title/Abstract]))OR(Unawareness States,Persistent[Title/Abstract]))OR(Permanent Vegetative State[Title/Abstract]))OR(Permanent Vegetative States[Title/Abstract]))OR(State,Permanent Vegetative[Title/Abstract]))OR(States,Permanent Vegetative[Title/Abstract]))OR(Vegetative State,Permanent[Title/Abstract]))OR(Vegetative States,Permanent[Title/Abstract]))OR(Vegetative State[Title/Abstract]))OR(State,Vegetative[Title/Abstract]))OR(States,Vegetative[Title/Abstract]))OR(Vegetative States[Title/Abstract]))OR(Post-Traumatic Vegetative State[Title/Abstract]))OR(Post Traumatic Vegetative State[Title/Abstract]))OR(Post-Traumatic Vegetative States[Title/Abstract]))OR(State,Post-Traumatic Vegetative[Title/Abstract]))OR(States,Post-Traumatic Vegetative[Title/Abstract]))OR(Vegetative State,Post-Traumatic[Title/Abstract]))OR(Vegetative States,Post-Traumatic[Title/Abstract]))OR(Posttraumatic Unawareness State[Title/Abstract]))OR(Posttraumatic Unawareness States[Title/Abstract]))OR(State,Posttraumatic Unawareness[Title/Abstract]))OR(States,Posttraumatic Unawareness[Title/Abstract]))OR(Unawareness State,Posttraumatic[Title/Abstract]))OR(Unawareness States,Posttraumatic[Title/Abstract]))OR(Post-Traumatic Unawareness State[Title/Abstract]))OR(Post Traumatic Unawareness State[Title/Abstract]))OR(Post-Traumatic Unawareness States[Title/Abstract]))OR(State,Post-Traumatic Unawareness[Title/Abstract]))OR(States,Post-Traumatic Unawareness[Title/Abstract]))OR(Unawareness State,Post-Traumatic[Title/Abstract]))OR(Unawareness States,Post-Traumatic[Title/Abstract]))OR(Prolonged Post-Traumatic Unawareness[Title/Abstract]))OR(Post-Traumatic Unawareness,Prolonged[Title/Abstract]))OR(Post-Traumatic Unawarenesses,Prolonged[Title/Abstract]))OR(Prolonged Post Traumatic Unawareness[Title/Abstract]))OR(Prolonged Post-Traumatic Unawarenesses[Title/Abstract]))OR(Unawareness,Prolonged Post-Traumatic[Title/Abstract]))OR(Unawarenesses,Prolonged Post-Traumatic[Title/Abstract]))OR(Transient Vegetative State[Title/Abstract]))OR(State,Transient Vegetative[Title/Abstract]))OR(States,Transient Vegetative[Title/Abstract]))OR(Transient Vegetative States[Title/Abstract]))OR(Vegetative State,Transient[Title/Abstract]))OR(Vegetative States,Transient[Title/Abstract]))OR(Minimally Conscious State[Title/Abstract]))OR(Minimally Conscious States[Title/Abstract]))OR(State,Minimally Conscious[Title/Abstract]))OR(States,Minimally Conscious[Title/Abstract]))OR(Post-Comatose Unawareness State[Title/Abstract]))OR(Post Comatose Unawareness State[Title/Abstract]))OR(Post-Comatose Unawareness States[Title/Abstract]))OR(State,Post-Comatose Unawareness[Title/Abstract]))OR(States,Post-Comatose Unawareness[Title/Abstract]))OR(Unawareness State,Post-Comatose[Title/Abstract]))OR(Unawareness States,Post-Comatose[Title/Abstract]))OR(Postcomatose Unawareness State[Title/Abstract]))OR(Postcomatose Unawareness States[Title/Abstract]))OR(State,Postcomatose Unawareness[Title/Abstract]))OR(States,Postcomatose Unawareness[Title/Abstract]))OR(Unawareness State,Postcomatose[Title/Abstract]))OR(Unawareness States,Postcomatose[Title/Abstract])))  (("Consciousness Disorders"[Mesh])OR((((((((Consciousness Disorder[Title/Abstract])OR(Disorders of Consciousness[Title/Abstract]))OR(Disorder of Consciousness[Title/Abstract]))OR(Consciousness,Level Depressed[Title/Abstract]))OR(Depressed Level of Consciousness[Title/Abstract]))OR(Semiconsciousness[Title/Abstract]))OR(Consciousness,Level Altered[Title/Abstract]))OR(Altered Level of Consciousness[Title/Abstract])))AND(("Median Nerve"[Mesh])OR(((Median Nerves[Title/Abstract])OR(Nerve,Median[Title/Abstract]))OR(Nerves,Median[Title/Abstract])))  (("Coma"[Mesh])OR((((Comas[Title/Abstract])OR(Comatose[Title/Abstract]))OR(Pseudocoma[Title/Abstract]))OR(Pseudocomas[Title/Abstract])))AND(("Median Nerve"[Mesh])OR(((Median Nerves[Title/Abstract])OR(Nerve,Median[Title/Abstract]))OR(Nerves,Median[Title/Abstract]))) |
| Cochrane | #1 MeSH descriptor:[Median Nerve]explode all trees 383  #2 (Nerves,Median or Median Nerves or Nerve,Median):ti,ab,kw(Word variations have been searched) 4304  #3 #1 OR#2 4304  #4 MeSH descriptor:[Persistent Vegetative State]explode all trees 84  #5 (States,Vegetative OR Vegetative State OR Vegetative States OR State,Vegetative OR Permanent Vegetative State OR Vegetative State,Permanent OR Permanent Vegetative States OR Vegetative States,Permanent OR State,Permanent Vegetative OR States,Permanent Vegetative OR Unawareness States,Post-Traumatic OR State,Post-Traumatic Unawareness OR Post-Traumatic Vegetative State OR Vegetative States,Post-Traumatic OR Unawareness State,Post-Traumatic OR States,Posttraumatic Unawareness OR States,Post-Traumatic Unawareness OR State,Post-Traumatic Vegetative OR Post-Traumatic Unawareness State OR Posttraumatic Unawareness States OR States,Post-Traumatic Vegetative OR Post-Traumatic Vegetative States OR Unawareness States,Posttraumatic OR Post Traumatic Vegetative State OR Posttraumatic Unawareness State OR Post-Traumatic Unawareness States OR Post Traumatic Unawareness State OR Vegetative State,Post-Traumatic OR Unawareness State,Posttraumatic OR State,Posttraumatic Unawareness OR Vegetative States,Persistent OR PVSs(Persistent Vegetative State)OR States,Persistent Unawareness OR State,Persistent Unawareness OR States,Persistent Vegetative OR Persistent Unawareness State OR Unawareness State,Persistent OR State,Persistent Vegetative OR Vegetative State,Persistent OR Unawareness States,Persistent OR Persistent Vegetative States OR Persistent Unawareness States OR PVS(Persistent Vegetative State)OR States,Minimally Conscious OR Minimally Conscious State OR Minimally Conscious States OR State,Minimally Conscious OR Prolonged Post-Traumatic Unawareness OR Unawarenesses,Prolonged Post-Traumatic OR Post-Traumatic Unawarenesses,Prolonged OR Unawareness,Prolonged Post-Traumatic OR Prolonged Post Traumatic Unawareness OR Post-Traumatic Unawareness,Prolonged OR Prolonged Post-Traumatic Unawarenesses OR States,Post-Comatose Unawareness OR Unawareness State,Postcomatose OR Postcomatose Unawareness States OR State,Post-Comatose Unawareness OR Unawareness States,Postcomatose OR Post-Comatose Unawareness State OR Unawareness States,Post-Comatose OR Unawareness State,Post-Comatose OR States,Postcomatose Unawareness OR Post-Comatose Unawareness States OR Postcomatose Unawareness State OR Post Comatose Unawareness State OR State,Postcomatose Unawareness OR State,Transient Vegetative OR Vegetative State,Transient OR States,Transient Vegetative OR Vegetative States,Transient OR Transient Vegetative States OR Transient Vegetative State):ti,ab,kw(Word variations have been searched) 1319  #6 #4 OR#5 1319  #7 #3 AND#6 12  #8 MeSH descriptor:[Consciousness Disorders]explode all trees 1385  #9 (Semiconsciousness OR Depressed Level of Consciousness OR Consciousness,Level Depressed OR Consciousness,Level Altered OR Altered Level of Consciousness OR Disorders of Consciousness OR Consciousness Disorder OR Disorder of Consciousness):ti,ab,kw(Word variations have been searched) 2582  #10 #8 OR#9 623172  #11 #10 AND#3 1783  #12 MeSH descriptor:[Coma]explode all trees 444  #13 (Pseudocomas OR Pseudocoma OR Comatose OR Comas):ti,ab,kw(Word variations have been searched) 4726  #14 #12 OR#13 4843  #15 #14 AND#3 35 |
| Medline | #Database:MEDLINE®  #Searches:  1:(((TS=(Median Nerve))OR TS=(Median Nerves))OR TS=(Nerve,Median))OR TS=(Nerves,Median)  Results:33255  2:(((((((((((((((((((((((((((((((((((((((((((((((((((((((((((((((((((((((((TS=(Persistent Vegetative State))OR TS=(PVS(Persistent Vegetative State)))OR TS=(PVSs(Persistent Vegetative State)))OR TS=(Vegetative State,Persistent))OR TS=(Persistent Vegetative States))OR TS=(State,Persistent Vegetative))OR TS=(States,Persistent Vegetative))OR TS=(Vegetative States,Persistent))OR TS=(Persistent Unawareness State))OR TS=(Persistent Unawareness States))OR TS=(State,Persistent Unawareness))OR TS=(States,Persistent Unawareness))OR TS=(Unawareness State,Persistent))OR TS=(Unawareness States,Persistent))OR TS=(Permanent Vegetative State))OR TS=(Permanent Vegetative States))OR TS=(State,Permanent Vegetative))OR TS=(States,Permanent Vegetative))OR TS=(Vegetative State,Permanent))OR TS=(Vegetative States,Permanent))OR TS=(Vegetative State))OR TS=(State,Vegetative))OR TS=(States,Vegetative))OR TS=(Vegetative States))OR TS=(Post-Traumatic Vegetative State))OR TS=(Post Traumatic Vegetative State))OR TS=(Post-Traumatic Vegetative States))OR TS=(State,Post-Traumatic Vegetative))OR TS=(States,Post-Traumatic Vegetative))OR TS=(Vegetative State,Post-Traumatic))OR TS=(Vegetative States,Post-Traumatic))OR TS=(Posttraumatic Unawareness State))OR TS=(Posttraumatic Unawareness States))OR TS=(State,Posttraumatic Unawareness))OR TS=(States,Posttraumatic Unawareness))OR TS=(Unawareness State,Posttraumatic))OR TS=(Unawareness States,Posttraumatic))OR TS=(Post-Traumatic Unawareness State))OR TS=(Post Traumatic Unawareness State))OR TS=(Post-Traumatic Unawareness States))OR TS=(State,Post-Traumatic Unawareness))OR TS=(States,Post-Traumatic Unawareness))OR TS=(Unawareness State,Post-Traumatic))OR TS=(Unawareness States,Post-Traumatic))OR TS=(Prolonged Post-Traumatic Unawareness))OR TS=(Post-Traumatic Unawareness,Prolonged))OR TS=(Post-Traumatic Unawarenesses,Prolonged))OR TS=(Prolonged Post Traumatic Unawareness))OR TS=(Prolonged Post-Traumatic Unawarenesses))OR TS=(Unawareness,Prolonged Post-Traumatic))OR TS=(Unawarenesses,Prolonged Post-Traumatic))OR TS=(Transient Vegetative State))OR TS=(State,Transient Vegetative))OR TS=(States,Transient Vegetative))OR TS=(Transient Vegetative States))OR TS=(Vegetative State,Transient))OR TS=(Vegetative States,Transient))OR TS=(Minimally Conscious State))OR TS=(Minimally Conscious States))OR TS=(State,Minimally Conscious))OR TS=(States,Minimally Conscious))OR TS=(Post-Comatose Unawareness State))OR TS=(Post Comatose Unawareness State))OR TS=(Post-Comatose Unawareness States))OR TS=(State,Post-Comatose Unawareness))OR TS=(States,Post-Comatose Unawareness))OR TS=(Unawareness State,Post-Comatose))OR TS=(Unawareness States,Post-Comatose))OR TS=(Postcomatose Unawareness State))OR TS=(Postcomatose Unawareness States))OR TS=(State,Postcomatose Unawareness))OR TS=(States,Postcomatose Unawareness))OR TS=(Unawareness State,Postcomatose))OR TS=(Unawareness States,Postcomatose)  Results:7742  3:#1 AND#2 Results:48  4:((((((((TS=(Consciousness Disorders))OR TS=(Consciousness Disorder))OR TS=(Disorders of Consciousness))OR TS=(Disorder of Consciousness))OR TS=(Consciousness,Level Depressed))OR TS=(Depressed Level of Consciousness))OR TS=(Semiconsciousness))OR TS=(Consciousness,Level Altered))OR TS=(Altered Level of Consciousness)  Results:13925  5:#1 AND#4 Results:36  6:((((TS=(Coma))OR TS=(Comas))OR TS=(Comatose))OR TS=(Pseudocoma))OR TS=(Pseudocomas)  Results:54746  7:#1 AND#6 Results:222 |
| CNKI, VIP, Wanfang. | Using the following search term (Median Nerves) , subject selection coma or Disorder of Consciousness . |

**S2 Table. Search strategy.**
