# Supplementary material for: Safety of median nerve electrical stimulation in disorders of consciousness: A systematic review and meta-analysis of randomized controlled trials
Source: PLoS One. 2025 Jul 31;20(7):e0324046. doi: 10.1371/journal.pone.0324046 (PMC12312889; doi:10.1371/journal.pone.0324046)
Supplement: S4 Table — (DOCX) [file pone.0324046.s004.docx]

**S4 Table. Characteristics of the included studies.**

|  | **First Author, year** | **Country** | **Trial design** | **Randomization method** | **Participant**  **(E/C)** | **Experimental**  **arm** | **Control**  **arm** | **Underlying**  **Disease()** | **Outcome** |
| --- | --- | --- | --- | --- | --- | --- | --- | --- | --- |
| 1 | Yanli Duan, 2023  Clinical observation on the treatment of chronic confusion by acupuncture method of awakening consciousness Combined with median nerve electrical stimulation | China | RCT | Random number table method | 40/40 | routine treatment + acupuncture + RMNS | routine treatment | pDoC | E：  a,b  C：  b |
| 2 | Yi Wang, 2022  Effects of Xingnaojing combined with median nerve electrical stimulation on coma patients with cerebral hemorrhage | China | RCT | Random number table method | 32/32 | routine treatment + MNS + Xingnaojing treatment | routine treatment | Coma caused by spontaneous intracerebral hemorrhage | E:  b,c |
| 3 | Xiaowu Cheng, 2022  Analysis on the efficacy of electrical stimulation of right median nerve on coma and awakening after craniocerebral injury | China | RCT | Random number table method | 30/30 | routine treatment + RMNS | routine treatment | Coma caused by craniocerebral injury | E：  f,g,h  C：  a,f,g,h |
| 4 | Kun Zhao, 2020  Effect of right median nerve stimulation on clinical efficacy and prognosis of patients  with consciousness disorder caused by traumatic brain injury | China | RCT | Random number table method | 46/46 | routine treatment + acupuncture + RMNS | routine treatment + acupuncture | DoC caused by cerebral TBI | E：  f,g,h  C：  f,g,h |
| 5 | Hanwen Huang, 2020  Effects of different electrical stimulation schemes on EEG activity and cerebral blood flow velocity in coma patients with cerebral hemorrhag | China | RCT | Random number table method | 44/44 | routine treatment + MNS | routine treatment + acupuncture (the pericardium meridian of hand jueyin) | Coma caused by cerebral hemorrhage | E：  b,d,e  C：  b,c,d,e |
| 6 | Zhenning Wang, 2019  Effects of median nerve electrical stimulation on cerebral blood flow velocity and cerebral blood flow in patients with coma after severe craniocerebral injury | China | RCT | Random number table method | 31/31 | routine treatment + RMNS | routine treatment | Coma caused by craniocerebral injury | E:  a,b,f,g  C：  f,g |
| 7 | Wanshun Wen, 2017  Clinical observation of the short-term arousal effect of early right median nerve electrical stimulation on coma patients with traumatic  brain injury | China | RCT | It only mentions random allocation, but does not mention the method of random allocation used. | 59/60 | routine treatment + RMNS | routine treatment | Coma caused by TBI | E：  a,b,f,g  C：  a,b,f,h |
| 8 | Zhidi Li, 2016  Effects the changes of naloxone hydrochloride combined with median nerve  electrical stimulation on cerebral blood flow and electrophysiological in patients  with cerebral hemorrhage coma | China | RCT | The method of drawing lots. | 33/33 | routine treatment+ naloxone hydrochloride + MNS | routine treatment + naloxone hydrochloride | Coma caused by cerebral hemorrhage | E：  c,d,e  C：  b,d |
| 9 | Sumin Huang, 2015  Observation and nursing of electrical stimulation of right median nerve on coma in patients with craniocerebral injury | China | RCT | Random number table method | 40/40 | routine treatment + RMNS | routine treatment | Coma caused by craniocerebral injury | E:  b |
| 10 | Jing Sun 2021  Clinical Effect Analisis of Right Median Nerve Electrical Stimulation on Early Coma  Patients With Craniocerebral Injury | China | RCT | It only mentions random allocation, but does not mention the method of random allocation used. | 40/40 | routine treatment + RMNS | routine treatment | coma caused by craniocerebral injury | E：  a,b,f,g,h  C：  b,f,h |
| 11 | Ting Gao 2021  The Study of Median Nerve Electric Therapy Instrument on Awakening Treatment of Elderly Patients with  Cerebral Hemorrhage | China | RCT | Random number table method | 45/45 | routine treatment + MNS | routine treatment + acupuncture (the pericardium meridian of hand jueyin) | Coma caused by cerebral hemorrhage | E：  b,d,e  C：  b,c,d,e |
| 12 | Xiaohong Wei 2017  Effect of Right Median Nerve Stimulation on the Recovery of Coma Patients with Traumatic Brain Injury | China | RCT | Random number table method | 41/41 | routine treatment + RMNS | routine treatment | Coma caused by TBI | E:  f,g,h  C：  f,g,h |
| 13 | Xiang Wu 2023  Acute traumatic coma awakening by right median nerve electrical stimulation: a randomised controlled trial |  | RCT | Stratified by study centre using a block randomisation scheme with a block size of 4. | 167/162 | routine treatment + RMNS | routine treatment | Coma caused by TBI | E：  a,f,h,g  C：  a,f,h,g |
| 14 | Jin Lei 2015  Right Median Nerve Electrical Stimulation for Acute Traumatic Coma Patients | China | RCT | Patients are assigned according to the date of their birth month.Those with an odd-numbered birth date in the month are assigned to the MNS group,and those with an even-numbered birth date are classified into the control group. | 221/216 | routine treatment + RMNS | routine treatment | Coma caused by TBI | E：  a,b,f,h  C：  a,b,f,h |

E: experimental group; C: control group; a: seizure; b: increased sympathetic activity; c: arrhythmia; d: nausea and vomiting; e: lethargy; f: pulmonary infection; g: intracranial hemorrhage or hematoma; h: gastrointestinal hemorrhage. TBI, traumatic brain injury. MNS, median nerve electrical stimulation; pDoC, prolonged disorders of consciousness; RMNS, right median nerve electrical stimulation.

**S4 Table. Characteristics of the included studies.**
