# Supplementary material for: Safety of median nerve electrical stimulation in disorders of consciousness: A systematic review and meta-analysis of randomized controlled trials
Source: PLoS One. 2025 Jul 31;20(7):e0324046. doi: 10.1371/journal.pone.0324046 (PMC12312889; doi:10.1371/journal.pone.0324046)
Supplement: S5 Table — (DOCX) [file pone.0324046.s005.docx]

**S5 Table. Quality of evidence.**

| **Certainty assessment** | | | | | | | **№ of patients** | | **Effect** | | **Certainty** | **Importance** |
| --- | --- | --- | --- | --- | --- | --- | --- | --- | --- | --- | --- | --- |
| **№ of studies** | **Study design** | **Risk of bias** | **Inconsistency** | **Indirectness** | **Imprecision** | **Other considerations** | **MNS** | **routine treatment** | **Relative (95% CI)** | **Absolute (95% CI)** |  |  |
| **Seizure** | | | | | | | | | | | | |
| 6 | randomised trials | serious | not serious | not serious | serious^a^ | none | 8/421 (1.9%) | 6/417 (1.4%) | **RR 1.22** (0.44 to 3.41) | **3 more per 1,000** (from 8 fewer to 35 more) | ⨁⨁◯◯ Low | IMPORTANT |
| **Increased sympathetic activity** | | | | | | | | | | | | |
| 10 | randomised trials | serious^b^ | not serious | not serious | serious^a^ | none | 25/585 (4.3%) | 29/581 (5.0%) | **RR 0.80** (0.46 to 1.38) | **10 fewer per 1,000** (from 27 fewer to 19 more) | ⨁⨁◯◯ Low | IMPORTANT |
| **Arrhythmia** | | | | | | | | | | | | |
| 4 | randomised trials | serious^b^ | not serious | not serious | serious^a^ | none | 3/154 (1.9%) | 2/154 (1.3%) | **RR 1.19** (0.25 to 5.67) | **2 more per 1,000** (from 10 fewer to 61 more) | ⨁⨁◯◯ Low | IMPORTANT |
| **Nausea and vomiting** | | | | | | | | | | | | |
| 3 | randomised trials | serious^b^ | not serious | not serious | serious^a^ | none | 4/122 (3.3%) | 9/122 (7.4%) | **RR 0.49** (0.15 to 1.64) | **38 fewer per 1,000** (from 63 fewer to 47 more) | ⨁⨁◯◯ Low | IMPORTANT |
| **Lethargy** | | | | | | | | | | | | |
| 3 | randomised trials | serious^b^ | not serious | not serious | serious^a^ | none | 3/122 (2.5%) | 2/122 (1.6%) | **RR 1.35** (0.26 to 7.04) | **6 more per 1,000** (from 12 fewer to 99 more) | ⨁⨁◯◯ Low | IMPORTANT |
| **Pulmonary infection** | | | | | | | | | | | | |
| 8 | randomised trials | serious^b^ | serious^c^ | not serious | serious^a^ | none | 144/635 (22.7%) | 101/626 (16.1%) | **RR 1.11** (0.62 to 2.00) | **18 more per 1,000** (from 61 fewer to 161 more) | ⨁◯◯◯ Very low | IMPORTANT |
| **Intracranial hemorrhage or hematoma** | | | | | | | | | | | | |
| 5 | randomised trials | serious^b^ | not serious | not serious | serious^a^ | none | 7/188 (3.7%) | 6/188 (3.2%) | **RR 1.13** (0.39 to 3.31) | **4 more per 1,000** (from 19 fewer to 74 more) | ⨁⨁◯◯ Low | IMPORTANT |
| **Gastrointestinal bleeding** | | | | | | | | | | | | |
| 7 | randomised trials | serious^b^ | not serious | not serious | serious^a^ | none | 26/604 (4.3%) | 22/595 (3.7%) | **RR 1.18** (0.66 to 2.10) | **7 more per 1,000** (from 13 fewer to 41 more) | ⨁⨁◯◯ Low | IMPORTANT |

CI: confidence interval; RR: risk ratio；a. 95% CI is wide；b. lack of blinding and the concealment of allocation are not clear；c. high heterogeneity including clinical heterogeneity or statistical heterogeneity.

**S5 Table. Quality of evidence.**
